# ASSOCIATIONS OF SOCIAL ISOLATION AND LONELINESS WITH MCI AMONG OLDER ADULTS FROM UNDERRESOURCED COMMUNITIES

**DOI:** 10.1093/geroni/igae098.0473

**Published:** 2024-12-31

**Authors:** Fang Fang, Tiffany Hughes, Yueting Wang, Erin Jacobsen

**Affiliations:** Old Dominion University-EVMS, Norfolk, Virginia, United States; Midwestern University, Glendale, Arizona, United States; University of Pittsburgh, Pittsburgh, Pennsylvania, United States; University of Pittsburgh, Pittsburgh, Pennsylvania, United States

## Abstract

Previous studies have shown social isolation and loneliness are associated with poor cognitive outcomes in later life, yet it is unclear if these findings could be confounded by selective attrition. This study examines longitudinal associations of social isolation and loneliness with incident mild cognitive impairment (MCI) accounting for informative drop-out due to death or disease. Data were from a population-based cohort study of older adults in Southwestern Pennsylvania, whose once-vibrant economy heavily relied on the steel industry until it collapsed in the 1970s and has not fully recovered. Participants (N=1,254) were followed for 12 years for the development of MCI. Composite measures of social isolation and loneliness were created using longitudinal data across the study period. A generalized linear mixed model (GLMM) analyzing incident MCI as a binary outcome with joint modeling to adjust for potential attrition bias, along with adjustment for demographic, health, and lifestyle factors was used to examine the associations of social isolation and loneliness with incident MCI. The analyses revealed that greater social isolation was associated with higher risk of incident MCI (odds ratio=1.33, p=0.014) even when accounting for potential attrition bias, other potentially confounding factors, and loneliness. Loneliness was not significantly associated with incident MCI with or without accounting for social isolation (odds ratio=1.04, p=0.716; odds ratio=1.12, p=0.255). Over time, social isolation, rather than loneliness, may negatively affect cognition and strategies to keep older adults socially connected may reduce the risk for cognitive impairment.

